# Exposure of Buffalo Milkers to Pathogenic Bacteria and Characterization of Isolated Methicillin-Resistant *Staphylococcus* spp.

**DOI:** 10.3390/ijerph19074353

**Published:** 2022-04-05

**Authors:** Federica Carraturo, Maria Chiara Alterisio, Jacopo Guccione, Valeria Cerullo, Michela Salamone, Michela Morelli, Giovanni Libralato, Ernesto Russo, Raffaele d’Angelo, Paolo Ciaramella, Antonio Di Loria, Marco Guida

**Affiliations:** 1 Hygiene Laboratories: Water, Food, Environment, Department of Biology, University of Naples Federico II, Via Cinthia 26, 80126 Naples, Italy; federica.carraturo@unina.it (F.C.); valery.cerullo.94.vc@gmail.com (V.C.); michela.salamone@unina.it (M.S.); michelamorelli1993@gmail.com (M.M.); giovanni.libralato@unina.it (G.L.); marco.guida@unina.it (M.G.); 2 Hygiene Laboratory, Centro Servizi Metrologici e Tecnologici Avanzati (CeSMA), University of Naples Federico II, Corso Nicolangelo Protopisani, 80146 Naples, Italy; 3 Department of Veterinary Medicine and Animal Production, University of Naples Federico II, Via Delpino 1, 8013 Naples, Italy; mariachiara.alterisio@unina.it (M.C.A.); paolo.ciaramella@unina.it (P.C.); 4 INAIL, Istituto Nazionale per L’assicurazione Contro Gli Infortuni Sul Lavoro, Direction of Regione Campania, Consulenza Tecnica Accertamento Rischi e Prevenzione-CONTARP, Via S. Nicola alla Dogana 9, 80133 Naples, Italy; er.russo@inail.it (E.R.); r.dangelo@inail.it (R.d.)

**Keywords:** buffalo, milking parlors, *Staphylococcus aureus*, zoonosis, occupational health, methicillin-resistant, MRSA

## Abstract

The research was focused on the surveillance of the exposure of buffalo milkers in contact with both animals and potentially contaminated equipment, pointing attention on the diffusion of antibiotic-resistant *Staphylococcus* spp. The monitoring was performed for 12 months, allowing the collection of 600 raw milk and buffalo udder surface samples, 192 milking lanes, 400 milking clusters, 160 personal protective equipment (PPEs) and electronic devices surface samples in contact with the workers of four milking parlors located in Southern Italy. The analysis of the milk samples evidenced the highest exposure to the bacteria considered (and mainly to *S. aureus*) from late winter–spring seasons onward. The possible risk arising from buffalo udder, milking clusters, and lines were instead considered rather stable along the entire period of sampling. The PPEs turned out to be a source of contamination for milkers mainly during the spring and summer periods. The analysis for oxacillin/methicillin resistance revealed in all the farms enrolled an overall amount of 37.5% of Staphylococci strains (belonging to *S. aureus, S. haemolyticus, S. pseudintermedius, S. chromogenes* species) resistant both to methicillin and oxacillin. The investigation demonstrated that the potential transfer of pathogenic bacteria to humans would have a better chance to occur at milk resumption time (since late winter–spring onward) when the number of animals to be milked is greater and the activity in the milking parlor is more challenging. At the same time, the findings seem to point out that the potential risk may be worsened by a significant presence of oxacillin/methicillin-resistant Staphylococci, potentially resulting from irrational use of antibiotics.

## 1. Introduction

The breeding of Mediterranean water buffalo (*Bubalus bubalis*) represents one of the most important agri-food sectors in Italian business, bringing a turnover of 477 million euros and an estimated production volume at over 1 billion litres in 2017 [1]. Buffaloes supply chain has widely developed thanks to its main food product, the buffalo mozzarella cheese, an essential resource mainly for Southern Italy, with a buffalo population of ~400,000 heads (95% of European population), providing over 15,000 jobs [2,3]. As recently reported in the literature, Italy declared production of buffalo milk exceeding two million tons, 85% produced by Regione Campania, thanks to the presence of 1297 breeding, predominantly located in Caserta and Salerno districts [2,4]. Considering the elevated amount of work units overall employed in the buffalo supply chain, it is essential to focus attention on the need to develop updated regulations and guidelines that, on health and safety matters, may be able to preserve the workers of the sector. To date, accurate analyses of the potential risks for buffalo milkers are indeed not yet available. In the absence of buffaloes’ tailored scientific data, information from bovine breeding systems is used, often resulting in nonreproducible and poorly useful outcomes.

Furthermore, the milking procedures in buffalo farms, when adequately codified and executed, constitute an element able to ensure and preserve animal health, the safety and quality of produced milk, and the safety of the operators [5,6,7], as already established by the Italian Legislative Decree 81/2008, European Directive 89/391/EEC (the so-called occupational safety and health, OSH, “Framework Directive”), and US OSHA Regulation 29 CFR 1975 [8,9,10].

As widely demonstrated [6,7,8,9,10,11], the use of incorrect preparing procedures for buffaloes’ udders can predispose to mastitis: an inflammatory process originating from the wrong interaction between the udders’ immunity, milking procedures, and microbiologic pressures (mainly provided by contagious bacteria) [1,12]. Furthermore, when an udder develops mastitis, the total microbial load of milk exponentially increases [1,11], exposing the employees of milking parlors to a higher risk of contracting a zoonosis (e.g., the infections caused by *Staphylococcus aureus*, *Escherichia coli*, *Klebsiella* spp., Mycoplasms, etc.), able to cause severe pathologies [13,14]. In September 2021, the European Food Safety Authority (EFSA) identified *Escherichia coli* and *Staphylococcus aureus* as the most relevant antimicrobial-resistant bacteria in cattle in the EU, with a ≥66% certainty [15], leading to point attention to such microorganisms in livestock environments. 

*Staphylococcus aureus* is one of the principal pathogens causing mastitis in dairy buffalo [2,12,16,17]. Among *Staphylococcus aureus* strains, methicillin-resistant *Staphylococcus aureus* (MRSA) is retained the most common causative agent of human infections in hospital and community reservoirs: the World Health Organization (WHO) listed MRSA as a high-priority bacterium needing deep research and insights for its hazard profile [18]. In livestock environments, when MRSA strains cause subclinical infections, it may be transferred to milk without alterations of sensory characteristics of the food products, thus spreading through the dairy chain. Many reports describe the prevalence of MRSA in bovine milk [19], and the transmission to the employees of farms and milking parlors [20], nevertheless, scarce information is present about this possibility in dairy Mediterranean buffalo breeding systems. MRSA emergence in livestock has been related to the excessive use of growth promoters and antimicrobials as preventive measures to reduce infections [21,22]. *Staphylococcus aureus* therefore developed resistance to β-lactam antibiotics, by acquiring the *Staphylococcal Cassette Chromosome* (SCC) *mecA* gene, which is able to modify the penicillin-binding protein (PBP2) [21,23]. The higher prevalence of MRSA in milk was reported from research linking the increase of antibiotic-resistant strains to lacking hygienic measures, such as the incorrect use of a pre- and post-milking udder dip, gloves, and other personal protective equipment (PPEs) employed for milking [24]. The main concern on the public health risk of MRSA from livestock is related to the spreading of resistance genes from dairy species to humans through the food chain [25].

In this context, our hypothesis was that buffalo milkers could be exposed to pathogenic bacteria during their daily activity, and their risk may be worsened by the possibility to be infected by MRSA strains. Considering the premises, the goal of the manuscript was (i) to observe the different type of microorganisms present in buffalo milk, milking clusters, parlors, personal protective equipment, and smartphones of milkers along the year; (ii) to characterize the *Staphylococcus* spp. strains isolated in order to identify methicillin resistance species.

## 2. Materials and Methods

### 2.1. General and Ethical Animal Care

From a sample of 60 Mediterranean buffalo farms randomly selected from Caserta and Salerno districts (Regione Campania, Italy), four of them were identified employing nonprobability convenience sampling. 

The enrolled farms respected the following eligibility criteria: (i) an artificially induced seasonal calving herd (late winter–springtime); (ii) a housing and overall management system respecting the minimum welfare standard; (iii) a regular mastitis-monitoring program (e.g., including regular SCC monitoring, data analysis, etc.), (iv) Mediterranean buffaloes milked twice a day, (v) analogous milking parlors and procedures, as well as number of milkers (*n* = 2).

The study was performed in two consecutive years. The latter received institutional approval from the Ethical Animal Care and Use Committee of the University of Naples Federico II (n.PG/2017/0099607). The common good clinical practices have been respected during the entire procedure [26]; moreover, the farmers were previously informed and in agreement with the purposes of the study.

### 2.2. Farms and Sampling

Two Mediterranean buffalo farms have been identified within Caserta and Salerno districts each. They were characterized by approximately 800 animals in each one. Samplings was performed for 12 months, covering 4 seasons, starting from November 2019 until October 2020 (i.e., autumn, winter, spring, and summer), conducting 16 sampling sessions (i.e., four per season). 

The monitoring allowed the collection of 608 quarter milk samples (*n* = 152/farm) and 608 samples from the skin surface of the corresponding quarter; 192 milking lanes surface samples (*n* = 12 samples/season/farm); 400 milking cluster surface specimens (i.e., shell, short milk tube, and claw *n* = 25 samples/season/farm); 96 PPEs (surface samples) used by the milkers during the working time (i.e., gloves, coats, and boots; *n* = 3 samples/milkers/season/farm); 32 surface samples of the smartphones belonging to milkers (*n* = 1 samples/milkers/season/farm). All the sampling was performed in the middle of the milking procedure. 

The collected samples, aiming at assessing the potential exposure to pathogenic bacteria, were analyzed employing microbiological analysis for the qualitative and quantitative research of the main safety and quality indicators for the matrices sampled in the study: Total Bacterial Count (TBC), *Escherichia coli* (*E. coli*), Enterococci, and *Staphylococcus aureus* (*S. aureus*). Whereas *S. aureus* strains were isolated, typical colonies underwent molecular analysis for strain identification.

### 2.3. Raw Milk Samples Monitoring

#### 2.3.1. Total Bacterial Count (TBC), *E. coli*, and Enterococci Analysis

According to ISO 4833-1:2013 (Total Bacterial Count, TBC), ISO 16649-1:2018 (*E. coli*), and ISO 21528-2:2017 (Enterococci), an aliquot of 10 mL of milk sample and 90 mL of 0.9% NaCl solution (Oxoid, Thermo Fisher Scientific, Waltham, MA, USA) were added to a Stomacher bag, subsequently homogenized using a Stomacher. Ten-fold serial dilutions were prepared, and 1 mL aliquots were pour-plated on nonselective Plate Count Agar (Total Bacterial Count), selective Tryptone Bile X-GLUC Agar (*E. coli*), and selective Slanetz and Bartley Agar (Enterococci) (Oxoid, Thermo Fisher Scientific, Waltham, MA, USA). Plate Count Agar plates were incubated at 30 ± 1 °C for 24 h [27]; Slanetz and Bartley Agar plates were incubated at 37 ± 1 °C for 24 h [28], Tryptone Bile X-GLUC Agar plates were incubated at 44 ± 1 °C for 24 h [29]. Total Bacterial Count, *E. coli*, and Enterococci analyses were quantitative: the colonies were counted, and the results were expressed in CFU/mL.

#### 2.3.2. *Staphylococcus* spp. and Staphylococcus Aureus Analysis

Aliquots of 10 mL milk samples and 90 mL Buffered Peptone Water (BPW—Oxoid, Thermo Fisher Scientific, Waltham, MA, USA) were homogenized in Stomacher bags, and 100 µL aliquots were spread plated on Baird Parker selective agar (Oxoid, Thermo Fisher Scientific, Waltham, MA, USA); agar plates were incubated at 37 ± 1 °C for 24 to 48 h. The presence of typical colonies was verified after 24 and 48 h. *S. aureus* colonies were recognizable observing a double halo, an inner opaque, and an outer transparent, on selective Baird Parker medium. Black colonies, without the visualization of halos, had to be counted separately and classified as *Staphylococcus* spp. (ISO 6888-1:2021) [30]. *S. aureus* characteristic colonies were isolated from Baird Parker agar plates and underwent subsequent characterization employing molecular microbiology methodologies and methicillin resistance evaluation using selective agarized medium.

### 2.4. Surface Samples’ Microbiological Monitoring

For the analysis of buffalo udder samples and the surfaces monitored in the present study (milking clusters, milking lanes, PPEs, and smartphones), specimens were collected using sterile swabs, sampling ca. 100 cm^2^ areas [31], gently rotating the swab across the sampling area. Specimens underwent microbiological analyses, according to ISO (*International Standards Organization*) standardized methodologies. The monitoring consisted in the evaluation of Total Bacterial Count at 30 °C (ISO 4833-1:2013) and the research of *Escherichia coli* (ISO 16649-1:2018), Enterococci (ISO 21528-2:2017), and *Staphylococcus aureus* (ISO 6888-1:2021). Swabs were homogenized in sterile 15 mL tubes containing 0.9% NaCl solution (Oxoid, Thermo Fisher Scientific, Waltham, MA, USA) [30]; tubes were mixed for 30 s using a vortex, and a 100 μL aliquot was spread-plated on nonselective Plate Count Agar (Total Bacterial Count), selective Tryptone Bile X-GLUC Agar (*E. coli*), selective Slanetz and Bartley Agar (Enterococci), and Baird Parker selective agar (*Staphylococcus aureus*) (Oxoid, Thermo Fisher Scientific, Waltham, MA, USA). Plates were incubated according to the specific ISO methodologies; colonies were counted, and the results were expressed in CFU/cm^2^ [27,28,29,30,31]. Coagulase-positive *Staphylococcus* spp. typical colonies were isolated from Baird Parker agar plates only from buffalo udder surface samples and underwent subsequent molecular and antibiotic resistance characterization.

### 2.5. Isolation and Molecular Characterization of Coagulase-Positive Staphylococcus *spp.* Colonies 

*Staphylococcus aureus* colonies isolated from buffalo udder surface samples and from milk specimens underwent DNA extraction: colonies were picked up and suspended in 70 μL Milli-Q Type 1 Ultrapure Water. Total DNA was extracted through denaturation for 10 min at 98 °C: samples were centrifuged samples at 8,000 rpm for 5 min at 4 °C, and supernatant was recovered. PCR reactions were carried out in a TECHNE Prime Thermal Cycler employing universal primers, with a 700 bp amplicon size, complementary to the 16S rRNA gene’s conserved V3–V6 regions [32,33]. The selected oligos were synthetized by Biofab Research srl, and PCR reactions were carried out according to Carraturo et al. (2021) [32]. Following the analysis of the amplified PCR products on 1.5% agarose gel, stained with GelRed (Nucleic acid gel stain, BIOTIUM), using a 100 bp DNA ladder as reference, the samples were shipped to an external service (Biofab Research srl, Rome, Italy) for purification and sequencing steps. To identify the strains, obtained sequences were compared to the NCBI Sequences Database [32,34].

### 2.6. Identification of Oxacillin/Methicillin-Resistant Staphylococcus *spp.* (MRSA) Strains

Oxacillin Resistance Screening Agar Base Selective Supplement (ORSAB—Oxoid, Thermo Fisher Scientific, Waltham, MA, USA), supplemented with Oxacillin Resistance Screening Agar Base Selective Supplement (ORSAB Selective Supplement—Oxoid, Thermo Fisher Scientific, Waltham, MA, USA) was employed to identify and characterize antibiotic-resistant *Staphylococcus aureus* strains.

The selective medium exploits aniline blue to demonstrate mannitol fermentation of Staphylococci, further consenting the presumptive identification of oxacillin/methicillin-resistant *S. aureus* (MRSA) strains. Presumptive *S. aureus* colonies, morphologically identified using the Baird Parker selective agar medium, were isolated and homogenized in 10 mL Tryptone Soya Broth (TSB—Oxoid, Thermo Fisher Scientific, Waltham, MA, USA), Aliquots of 100 mL each of strain culture were spread-plated on ORSAB plates and incubated at 37 ± 1 °C for 24 h. MRSA strains were identified visualizing the typical intense blue colonies on selective medium, because of the aniline blue substrate [35].

### 2.7. Statistical Analysis

Collected data were evaluated statistically and graphically using Microsoft Office Excel (Microsoft Corporation, Redmond, WA, USA). The results presented refer to the arithmetic means for each type of sample collected (e.g., milk, udder, milking cluster, PPE, etc.) at every milking parlor. Standard deviation (σ) and standard error of the mean (SE), according to standard formulas, were additionally calculated; standard error bars are represented on the bars of each figure (values are showed as the mean ± standard error).

## 3. Results and Discussion

### 3.1. Microbiological Analysis

Of *n* = 608 milk samples, *n* = 8 were considered as contaminated (1.3%), because more than three dissimilar colonies were found as suggested by Guccione et al. (2014) [36]. The sterile swabs collected from the corresponding Mediterranean buffalo’s udder skin quarter were also discharged as a consequence.

The analysis of milkers’ exposure evidenced the isolation of pathogenic microorganisms potentially transferrable to humans, and therefore allowed to determine the onset of various types of zoonosis and pathologies of different severity levels. Among the microorganisms recognized as a cause of mastitis in buffaloes and responsible of human zoonosis [15], the results brought out the characterization of different strains of *Staphylococcus* spp.

**Figure 1:**
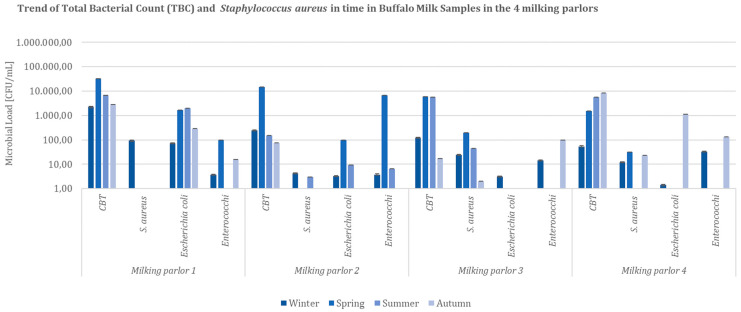
Trend of Total Bacterial Count (TBC), *Staphylococcus aureus, Escherichia coli*, and Enterococci load in time in buffalo milk samples collected from the 4 buffalo milking parlors involved in the study (CFU = Colony Forming Unit; cm^2^ = sampled surface; CBT = Total Bacterial Count).

Milk samples’ data evaluation allowed the seasonal comparison of the total bacterial count (TBC) values and coagulase-positive *Staphylococcus* spp., *E. coli*, and Enterococci microbial loads. Analysis of the results presumably evidenced a potential risk for workers to contract a zoonosis (mainly connected to *S. aureus* infections) predominantly during the winter and spring seasons (Figure 1). The highest microbial load values for TBC, contamination indicators, and coagulase-positive *Staphylococcus* spp. were registered in Milking Parlor 1 (MP1). It was indeed evident that the actions pointed at reducing the exposure for milking parlors’ workers should be primarily taken mainly during the two seasons resulted in the evaluation (Figure 1).

**Figure 2:**
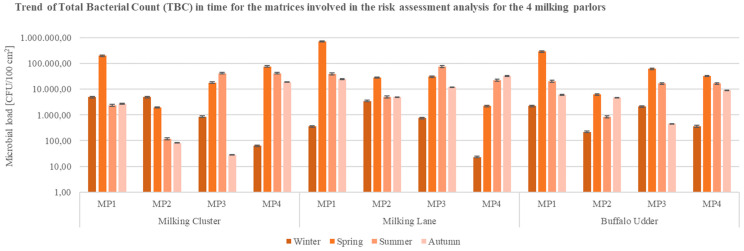
Trend of Total Bacterial Count (TBC) in time for the matrices (milking cluster, milking lane, buffalo udder) involved in the analysis for the 4 buffalo milking parlors under analysis (CFU = Colony Forming Unit; cm^2^ = sampled surface; MP = Milking Parlor).

The analysis also involved the exposure to bacteria for milking parlors’ employees through contact with the buffaloes, and the matrices with whom animals were in contact (milking clusters, milking lanes, and buffalo udders) (Figure 2): TBC values from Milking Parlor 1 (MP1) were higher (>10^5^ CFU/100 cm^2^) than those evidenced in MP2, MP3, and MP4 (<10^5^ CFU/100 cm^2^), especially during the spring season, while the trend decreased in the summer and autumn of 2020. Nonetheless, European guidelines, Regulation (EC) No 853/2004, set the limits of total bacterial count (at 30 °C) of raw milk from bovine species, excluding cow milk, at a range between 500,000 and 1,5 million CFU/mL [37].

**Figure 3:**
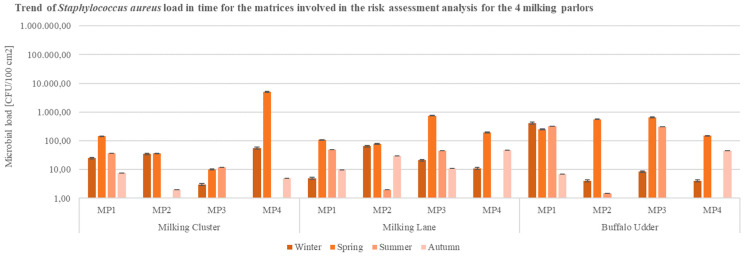
Trend of coagulase-positive *Staphylococcus* spp. (*S. aureus*) load in time for the matrices (milking cluster, milking lane, buffalo udder) involved in the analysis for the 4 buffalo milking parlors under analysis (CFU = Colony Forming Unit; cm^2^ = sampled surface; MP = Milking Parlor).

Thus, as well as with milk samples, total microbial load values higher than 10^4^ CFU/100 cm^2^ were registered in the surface samples of equipment and udders of buffaloes involved in the MP1 (Figure 2), in some cases corresponding to a higher isolation rate of coagulase-positive *Staphylococcus* spp. (Figure 3). As for milking clusters, the highest microbial loads of coagulase-positive *Staphylococcus* spp. (*Staphylococcus aureus*) were evidenced in MP4; elevated values of the pathogen were registered in the samples collected from the milking lanes of MP3 and MP4, mostly in the spring season (Figure 3).

During spring season, high TBC values correspond to the highest finding of coagulase-positive *Staphylococcus* spp. (*S. aureus*), in samples of udder, milking clusters, and lanes collected from the four milking parlors: it is indeed essential to specify that sampling for the spring season was performed at the ending period of the spring season (May–June 2020), allowing to presume that the rise of temperature may have led to the increase of pathogenic microbial load. Furthermore, coagulase-positive *Staphylococcus* spp. values exceeded 10^4^ CFU/100 cm^2^ exclusively in milking clusters samples from MP4; specimens of udder, milking clusters, and milking lanes from other milking parlors, in the period under analysis, did not overcome 10^3^ CFU/100 cm^2^ values (Figure 3). Total microbial counts and coagulase-positive *Staphylococcus* spp., following the monitoring of the four milking parlors, resulted in a decrease along the seasons, registering the lowest microbial counts during the autumn season (10^2^ CFU/100 cm^2^) (Figure 3).

**Figure 4:**
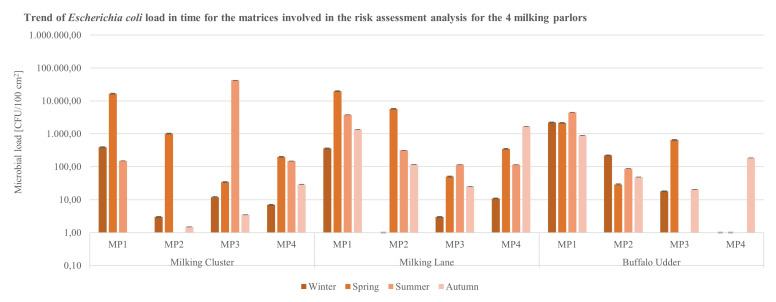
Trend of *E. coli* load in time for the matrices (milking clusters, milking lane, buffalo udder) involved in the analysis for the 4 buffalo milking parlors under analysis (CFU = Colony Forming Unit; cm^2^ = sampled surface; MP = Milking Parlor).

**Figure 5:**
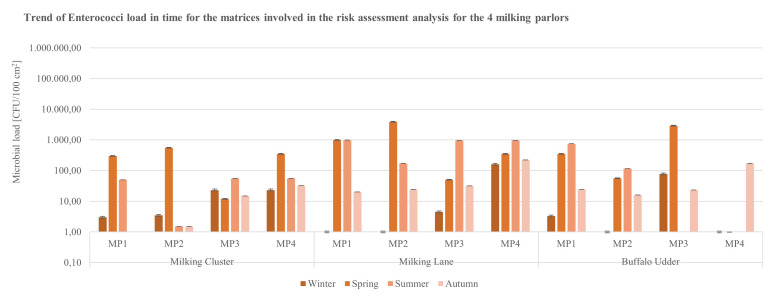
Trend of Enterococci load in time for the matrices (milking cluster, milking lane, buffalo udder) involved in the analysis for the 4 buffalo milking parlors under analysis (CFU = Colony Forming Unit; cm^2^ = sampled surface; MP = Milking Parlor).

Data for the contamination indicators *E. coli* (Figure 4) and Enterococci (Figure 5), with regards to the matrices in contact with the animals (milking clusters and milking lanes), reported microbial loads of the parameters higher than 10^3^ CFU/100 cm^2^ exclusively on milking clusters’ specimens sampled in MP4 during the spring season (Figure 4).

**Figure 6:**
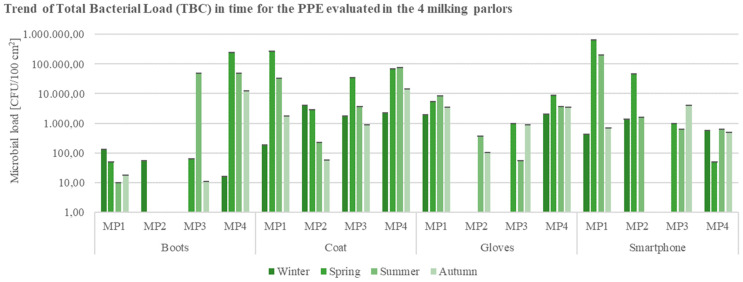
Trend of Total Bacterial Count (TBC) in time for the Personal Protection Equipment (PPEs: boots, coat, gloves) and smartphones evaluated in the 4 buffalo milking parlors under analysis (CFU = Colony Forming Unit; cm^2^ = sampled surface; MP = Milking Parlor).

The evaluation of the workers’ safety in the milking parlors involved in the research, with respect to the monitoring of the PPEs during the assessment period, evidenced elevated microbial loads mainly during the summer and spring seasons. In particular, data regarding surface samples of boots, coats, and gloves from MP4, in the warmest seasons, brought out total bacterial counts values ≥10^4^ CFU/100 cm^2^ (Figure 6).

Results’ analysis on PPEs and smartphones’ surface specimens described the highest microbial loads in coats and smartphones, registering peaks in the spring season, mainly in the samples collected from MP1 (Figure 6).

**Figure 7:**
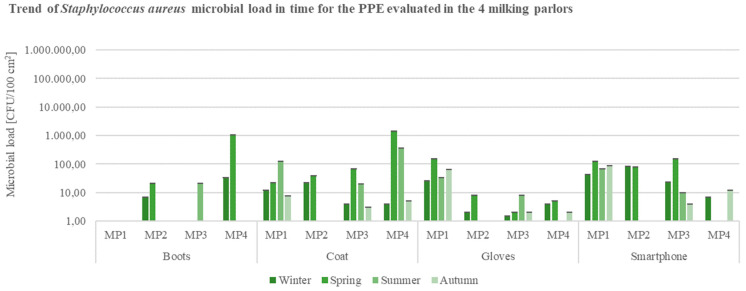
Trend of coagulase-positive *Staphylococcus* spp. (*S. aureus*) load in time for Personal Protection Equipment (PPE: boots, coat, gloves) and smartphones evaluated in the 4 buffalo milking parlors under analysis (CFU = Colony Forming Unit; cm^2^ = sampled surface; MP = Milking Parlor).

Coagulase-positive *Staphylococcus* spp. (*S. aureus*) evaluation allowed to suppose the identification of coats and smartphones as the most contaminated samples, with a substantial decrease in autumn in the totality of milking parlors. TBC and *S. aureus* counts of boots surface samples from MP1, MP3, and MP3 were the lowest registered: contamination indicators were mostly absent in the summer and winter (Figure 7).

**Figure 8:**
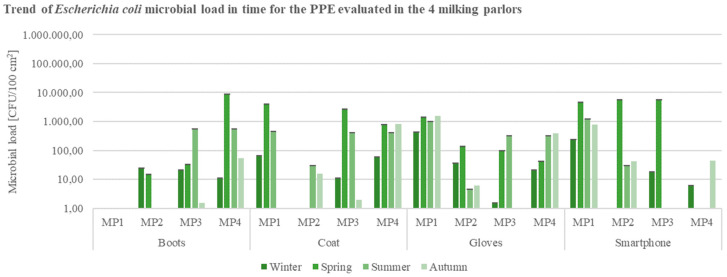
Trend of *E. coli* load in time for the Personal Protection Equipment (PPE: boots, coat, gloves) and smartphones evaluated in the 4 buffalo milking parlors under analysis (CFU = Colony Forming Unit; cm^2^ = sampled surface; MP = Milking Parlor).

Values of *E. coli* (Figure 8) for PPEs samples resulted higher than 10^3^ CFU/cm^2^ mainly in the spring season. Boots’ and coats’ surfaces of employees from MP3 and MP4 presented the highest loads of the indicator microorganism, with a substantial reduction in autumn. Gloves’ and smartphones’ surface specimens sampled from MP1 evidenced high microbial loads (Figure 8).

**Figure 9:**
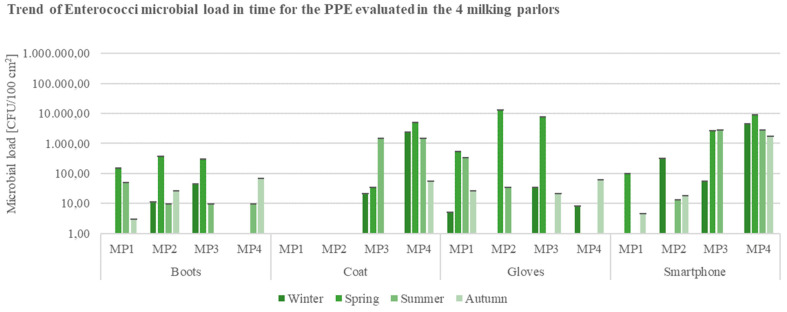
Trend of Enterococci load in time for the Personal Protection Equipment (PPEs: boots, coat, gloves) and smartphones evaluated in the 4 buffalo milking parlors under analysis (CFU = Colony Forming Unit; cm^2^ = sampled surface; MP = Milking Parlor).

The evaluation of PPEs and electronical devices for the parameter Enterococci (Figure 9) instead showed elevated microbial loads on coats and smartphone surfaces sampled from MP2 and MP3, in the spring season, confirming the need to intensify the sanitation protocols in the specific period.

### 3.2. Staphylococcus *spp.* identification and MRSA strains

Suspect *S. aureus* colonies (i.e., those developing a clear halo around the black colonies on Baird Parker agar, indicating positivity to coagulase), isolated from raw milk and milking clusters’ surface samples using selective agarized medium (Baird Parker selective agar, Oxoid, Thermo Fisher Scientific, Waltham, MA, USA) were subcultured and underwent molecular analysis for the identification of the strains.

Results of Sanger sequencing are shown in Table 1, Table 2, Table 3 and Table 4. A total of 32 colonies were characterized from the milking parlors: 14 strains from MP1 (Table 1), six from MP2 (Table 2), eight from MP3 (Table 3), four from MP4 (Table 4). Beyond *S. aureus* strains, *Staphylococcus epidermidis*, *Staphylococcus haemolyticus*, *Staphylococcus pseudintermedius*, *Staphylococcus jettensis, Staphylococcus muscae*, *Staphylococcus rostri*, *Staphylococcus chromogenes*, *Staphylococcus xylosus*, and *Staphylococcus hyicus* were identified. The analysis aimed to identify antibiotic-resistant Staphylococci: 37.5% strains (i.e., 12 isolates), belonging to species *S. aureus*, *S. haemolyticus*, *S. pseudintermedius*, *S. chromogenes* resulted resistant to methicillin and oxacillin, confirming the high potential risk for employees of contracting infections due to multiresistant Staphylococci. *S. aureus* strain cow, isolated from buffalo udder surface samples of MP1, was isolated in winter and further characterized in spring and summer, proving the inefficacy of mastitis treatments and sanitation protocols. The same strain was identified from buffalo udder surface samples of MP2 and MP3 in winter and spring (Table 5). 

It can be further hypothesized that the isolation of *S. aureus* strain cow from raw milk samples may depend on the transfer of pathogenic microorganisms from the udders of buffaloes that have been diagnosed infected to the food matrix.

The prevalence of methicillin-resistant *S. aureus* (MRSA) in buffalo dairy farms and raw milk was recently investigated by Normanno et al. (2020) [38] in Italy and Badua et al. (2020) [39] in the Philippines. Such prevalence of MRSA detected and characterized from buffalo and bovine milk samples was earlier reported worldwide, bringing out rates between 15% of isolated *S. aureus* (in China) [40], 41% (in Turkey) [41], and 61% (in the Philippines) [39]. The outcomes of the present study, 37.5% of isolated coagulase-positive Staphylococci (Table 5), can be placed within the mean values of available studies. *S. aureus* strain TPS3156 was reported as a methicillin-resistant *Staphylococcus* strain [42]. Noteworthy was the identification of oxacillin/methicillin-resistant non-aureus Staphylococci, such as *S. haemolyticus*, *S. pseudintermedius*, and *S. chromogenes*. *S. pseudintermedius* (strain 157588) was previously classified as a methicillin-resistant microorganism, isolated from dog skin, and responsible for infections in dogs [43,44]. *Staphylococcus intermedius*, phylogenetically similar to *S. pseudintermedius*, was isolated from water buffalo milk and dairy products in Turkey and was characterized as methicillin-resistant [45]. Furthermore, *S. pseudintermedius* shares 41% of identity with *S. chromogenes*, earlier described as coagulase-negative, afterwards demonstrated coagulase-positive *Staphylococcus* sp. [46]. *S. chromogenes* strain 34B, methicillin-resistant microorganism isolated from milk samples of Milking Parlor 2, was previously characterized from milk samples of buffaloes affected by subclinical mastitis [47].

Compared to cows, studies focusing on buffalo milking parlors are extremely rare, highlighting the need to intensify the monitoring of mastitis (e.g., Staphylococcal mastitis) onset, thus evaluating the transmission to livestock employees through environmental routes. Hence, the execution of the proper milking practices, the knowledge of potential sources of pathogenic microorganisms involved in the onset of harmful zoonoses, and the use of personal protective equipment (PPEs) nowadays represent the most efficient strategies able to reduce the biological risk in livestock companies in general, and in buffalo milking parlors in particular. The aim may be achieved through continuous and accurate information and formation phase addressed to livestock operators. The acquisition of enhanced awareness of the issues connected to the Mediterranean buffalo breeding system, as well as the measures aimed to provide information regarding potential biological risks for employees, represents the most optimized approach to obtain concrete results in terms of workers’ safety.

## 4. Conclusions

The surveillance of the practices of milking parlors’ employees represents the key to ensure the safety of the specific category of workers, exposed daily to several potential biological risks. According to the Legislative Decree 81/2008 of the Italian Parliament, the risk assessment activities of workers employed in milking parlors constitutes a legal obligation aimed at guaranteeing the safety of milk as a food product. In general, the conducted microbiological monitoring allows to confirm that the total bacterial count (TBC) results fall within the criteria indicated by Regulation (EC) No 853/2004 of the European Parliament (≤100.000 CFU/mL). *S. aureus*, as one of the main etiological agents of mastitis and the main cause of probable transfer of severe infections to the workers, must be accurately and timely monitored, mitigating the risk of infection caused by antibiotic-resistant strains. The observation of the trend of bacterial loads allowed to hypothesize that the highest bacterial exposure referred to the spring and summer seasons. During spring- and summertime, it would be optimal to intensify the monitoring sessions, the sanitation practices, strategies allowing the reduction of harmful microbial loads, beyond promoting the safety of workers. The employment of adequate milking procedures, the proper use of personal protective equipment, and the continuous education of the employees are essential to contain the biological risk to which milkers are exposed in the buffalo milking supply chain. Further studies quantifying the risk to which milkers and farm staff are exposed daily should be performed in order to suggest preventing strategies.

## Figures and Tables

**Table 1:** Molecular characterization, following Sanger sequencing, of *Staphylococcus* spp. strains isolated from buffalo milk samples (Milk) and buffalo udders surface swabs (Skin) collected in Milking Parlor 1 in the 4 seasons. Strains of *S. aureus* and methicillin-resistant *S. aureus* (MRSA) are indicated in **bold**. MRSA+ indicates the strain’s positivity to antibiotic resistance test towards methicillin.

Microorganism	Isolated from	Season	Max Score	Total Score	Query Cover	e-Value	% Identity	Accession n.	MRSA +
***Staphylococcus aureus* TPS3156**	**Milk**	Winter	**1.229**	**7.341**	**100%**	**0.0**	**99.85%**	**AP023034.1**	
*Staphylococcus epidermidis* strain 3039	Milk	Winter	1.264	1.264	100%	0.0	100.00%	MT613456.1	
***Staphylococcus aureus* strain AF7**	**Milk**	Winter	**1.264**	**1.264**	**99%**	**0.0**	**100.00%**	**MH645147.1**	**X**
*Staphylococcus haemolyticus* strain CPC	Milk	Winter	1.254	1.254	99%	0.0	100.00%	JX656750.1	
*Staphylococcus haemolyticus* strain MTCC 29970	Milk	Winter	1.225	1.225	99%	0.0	98.84%	MT569983.1	
*Staphylococcus* sp. strain f50-6	Milk	Winter	1.267	1.267	100%	0.0	99.86%	DQ837034.1	
***Staphylococcus aureus* TPS3156**	**Skin**	**Winter**	**1.256**	**7.529**	**100%**	**0.0**	**99.56%**	**AP023034.1**	
***Staphylococcus pseudintermedius* strain 157588**	**Skin**	**Winter**	**507**	**3.042**	**95%**	**2.00 × 10^−139^**	**95.11%**	**CP054206.1**	**X**
***Staphylococcus aureus* strain cow**	**Skin**	**Winter**	**1.277**	**1.277**	**100%**	**0.0**	**99.57%**	**MT628394.1**	**X**
*Staphylococcus jettensis* strain SEQ256	Skin	Winter	1.249	1.249	100%	0.0	99.14%	JN092114.2	
***Staphylococcus aureus* strain cow**	**Skin**	**Summer**	**1.277**	**1.277**	**98%**	**0.0**	**99.43%**	**MT628394.1**	**X**
*Staphylococcus muscae* strain PCM 2406	Milk	Spring	944	944	96%	0.0	96.99%	MF678949.1	
*Staphylococcus rostri* strain DSM 21968	Milk	Spring	1.886	1.886	97%	0.0	96.91%	MF678958.1	
***Staphylococcus aureus* strain cow**	**Skin**	**Spring**	**1.188**	**1.188**	**99%**	**0.0**	**97.42%**	**MT628394.1**	**X**

**Table 2:** Molecular characterization, following Sanger sequencing, of *Staphylococcus* spp. strains isolated from buffalo milk samples (Milk) and buffalo udders surface swabs (Skin) collected in Milking Parlor 2 in the 4 seasons. Strains of *S. aureus* and methicillin-resistant *S. aureus* (MRSA) are indicated in **bold**. MRSA+ indicates the strain’s positivity to antibiotic resistance test towards methicillin.

Microorganism	Isolated from	Season	Max Score	Total Score	Query Cover	e-Value	% Identity	Accession n.	MRSA +
***Staphylococcus chromogenes* strain 34B**	**Milk**	**Winter**	**1.286**	**7.657**	**99%**	**0.0**	**99.44%**	**CP031470.1**	**X**
***Staphylococcus aureus* strain cow**	**Skin**	**Winter**	**1.279**	**1.279**	**99%**	**0.0**	**99.71%**	**MT628394.1**	
***Staphylococcus aureus* strain cow**	**Milk**	**Winter**	**1.188**	**1.188**	**99%**	**0.0**	**97.42%**	**MT628394.1**	**X**
***Staphylococcus aureus* strain cow**	**Skin**	Spring	**1.293**	**1.293**	**99%**	**0.0**	**99.72%**	MT628394.1	
***Staphylococcus xylosus* strain AL173**	**Milk**	Summer	1.184	1.184	99%	0.0	98.94%	MG819270.1	
***Staphylococcus aureus* strain cow**	Milk	Summer	**1.120**	**1.120**	**94%**	**0.0**	**95.86%**	**MT628394.1**	

**Table 3:** Molecular characterization, following Sanger sequencing, of *Staphylococcus* spp. strains isolated from buffalo milk samples (Milk) and buffalo udders surface swabs (Skin) collected in the Milking Parlor 3 in the 4 seasons. Strains of *S. aureus* and methicillin-resistant *S aureus* (MRSA) are indicated in **bold**. MRSA+ indicates the strain’s positivity to antibiotic resistance test towards methicillin.

Microorganism	Isolated from	Season	Max Score	Total Score	Query Cover	e-Value	% Identity	Accession n.	MRSA +
*Staphylococcus* sp. strain H-MRSA-155a	Milk	Winter	1.146	1.146	96%	0.0	96.84%	KT153203.1	
***Staphylococcus haemolyticus* strain J-2-28**	Milk	Winter	**852**	**852**	**99%**	**0.0**	**99.36%**	MT312784.1	X
***Staphylococcus aureus* strain 61.1.3**	**Milk**	**Winter**	**675**	**675**	**98%**	**0.0**	**88.87%**	**KX454141.1**	**X**
*Staphylococcus haemolyticus* strain J-2-28	Skin	Winter	1.264	1.264	100%	0.0	99.57%	MT312784.1	
***Staphylococcus aureus* strain cow**	**Skin**	**Winter**	**1.267**	**1.267**	**99%**	**0.0**	**99.57%**	**MT628394.1**	
***Staphylococcus aureus* strain cow**	**Milk**	**Spring**	**1.282**	**1.282**	**99%**	**0.0**	**99.71%**	MT628394.1	
***Staphylococcus aureus* strain cow**	**Skin**	**Spring**	**1.286**	**1.286**	**99%**	**0.0**	**99.58%**	MT628394.1	
*Staphylococcus chromogenes* strain 34B	Milk	Spring	1.282	7.635	99%	0.0	99.71%	CP031470.1	

**Table 4:** Molecular characterization, following Sanger sequencing, of *Staphylococcus* spp. strains isolated from buffalo milk samples (Milk) and buffalo udders surface swabs (Skin) collected in Milking Parlor 4 in the 4 seasons. Strains of *S. aureus* and methicillin-resistant *S. aureus* (MRSA) are indicated in **bold**. MRSA+ indicates the strain’s positivity to antibiotic resistance test towards methicillin.

Microorganism	Isolated from	Season	Max Score	Total Score	Query Cover	e-Value	% Identity	Accession n.	MRSA +
***Staphylococcus aureus* strain MS1-4**	**Milk**	Winter	**1.271**	**1.271**	**99%**	**0.0**	**99.15%**	**LC378383.1**	**X**
***Staphylococcus aureus* strain TPS3156**	**Milk**	Winter	**1.266**	**7.563**	**100%**	**0.0**	**99.85%**	**AP023034.1**	**X**
***Staphylococcus aureus* strain 4726**	**Milk**	Winter	**1.247**	**1.247**	**100%**	**0.0**	**99.14%**	**MN923027.1**	**X**
*Staphylococcus hyicus* strain PCM 2192	Skin	Summer	538	538	64%	1.00 × 10^148^	93.72%	MF678941.1	

**Table 5:** Isolation in the different seasons of *S. aureus* strains isolated from buffalo milk samples (Milk) and buffalo udders surface swabs (Skin), collected in the 4 milking parlors monitored in the study.

Milking Parlor	Isolated from	*Staphylococcus aureus* isolate	Winter	Spring	Summer	Autumn
MP1	Buffalo Milk	*Staphylococcus aureus* strain TPS3156	X			
		*Staphylococcus aureus* strain AF7	X			
	Buffalo Udder	*Staphylococcus aureus* strain cow	X	X	X	
		*Staphylococcus aureus* strain TPS3156	X			
Milking Parlor	Isolated from	*Staphylococcus aureus* isolate	Winter	Spring	Summer	Autumn
MP2	Buffalo Milk	*Staphylococcus aureus* strain cow	X		X	
	Buffalo Udder	*Staphylococcus aureus* strain cow	X	X		
Milking Parlor	Isolated from	*Staphylococcus aureus* isolate	Winter	Spring	Summer	Autumn
MP3	Buffalo Milk	*Staphylococcus aureus* strain 61.1.3	X			
		*Staphylococcus aureus* strain cow		X		
	Buffalo Skin	*Staphylococcus aureus* strain cow	X	X		
Milking Parlor	Isolated from	*Staphylococcus aureus* isolate	Winter	Spring	Summer	Autumn
MP4	Buffalo Milk	*Staphylococcus aureus* strain MS1-4	X			
		*Staphylococcus aureus* strain TPS3156	X			
		*Staphylococcus aureus* strain 4726	X			

## Data Availability

Not applicable.
